# Development of multiplex real-time PCR assays for identification of members of the *Anopheles funestus *species group

**DOI:** 10.1186/1475-2875-8-282

**Published:** 2009-12-09

**Authors:** Samuel B Vezenegho, Chris Bass, Mirel Puinean, Martin S Williamson, Linda M Field, Maureen Coetzee, Lizette L Koekemoer

**Affiliations:** 1 Vector Control Reference Unit, National Institute for Communicable Diseases of the NHLS, Private Bag X4, Sandringham, Johannesburg 2131, South Africa; 2 Malaria Entomology Research Unit, School of Pathology of the University of the Witwatersrand and the National Health Laboratory Service, Johannesburg, South Africa; 3 Department of Biological Chemistry, Rothamsted Research, Harpenden, AL5 2JQ, UK

## Abstract

**Background:**

The malaria vector and non-vector species of the *Anopheles funestus *group are morphologically very similar and accurate identification is required as part of effective control strategies. In the past, this has relied on morphological and cytogenetic methods but these have been largely superseded by a robust allele-specific PCR (AS-PCR). One disadvantage of AS-PCR is the requirement for post-PCR processing by gel electrophoresis of PCR products. In this study, three new high-throughput 'closed-tube' assays were developed and compared with the previously described AS-PCR technique.

**Methods:**

Protocols for three fluorescence-based assays based on Melt Curve Analysis (MCA), High Resolution Melt (HRM) and TaqMan SNP genotyping were developed to detect and discriminate *Anopheles parensis, Anopheles leesoni, Anopheles vaneedeni, Anopheles rivulorum *and *An. funestus s.s*. The sensitivity and specificity of these assays were compared with the widely used AS-PCR in a blind trial using DNA extracted from wild-caught mosquitoes.

**Results:**

The TaqMan assay proved to be the most sensitive and specific of the three new assays. The MCA and HRM assays initially gave promising results, but were more sensitive to both DNA quality and quantity and consequently showed a higher rate of incorrect identifications.

**Conclusion:**

The TaqMan assay proved to be the most robust of the three protocols tested in this study. This assay very effectively identified all five members of the *An. funestus *group using fluorescently-labeled probes with distinct emission and excitation spectra allowing their independent detection in a single reaction. This method is at least as sensitive and specific as the gold standard AS-PCR approach and because it has no requirement for post-PCR processing is simpler and more rapid to run. The one disadvantage of the TaqMan assay is the cost of this assay, both in terms of initial capital outlay and running cost per sample, which is higher than AS-PCR. However, the cost of both the real-time PCR machine and fluorescently labelled probes required is falling and in the future the cost of this assay is likely to become closer to that of standard PCR.

## Background

The *Anopheles funestus *group consists of five subgroups of mosquitoes namely *Anopheles funestus, Anopheles rivulorum, Anopheles minimus, Anopheles aconitus *and *Anopheles culicifacies *and includes one of the most important African vectors of malaria *Anopheles funestus s.s. *[1-3]. The African mosquito species *An. funestus s.s., Anopheles parensis *and *Anopheles vaneedeni*, belonging to the *An. funestus *subgroup, and *Anopheles leesoni *and *Anopheles rivulorum*, belonging to the *An. minimus *and *An. rivulorum *subgroups, respectively, are either morphologically identical or very similar and may occur in sympatry over large parts of their distribution [3]. *Anopheles funestus s.s.*, *An. rivulorum *and *An. leesoni *have a widespread distribution across sub-Saharan Africa, while *An, parensis *is found through eastern and southern Africa and *An. vaneedeni *is restricted to South Africa [1,2].

The vectorial capacity, biology and behaviour of these species also differ. *Anopheles funestus s.s. *is an efficient vector of the human malaria parasite [2] and this is reflected by its anthropophilic and endophilic behaviour. The other species are predominantly zoophilic and exophilic [1,2] and are thought to have limited, or no, importance as malaria vectors, although *An. vaneedeni *has been shown to be susceptible to *Plasmodium *infection under laboratory conditions [4,5], and *An. rivulorum *has been found infected with the *Plasmodium *parasite at one locality in Tanzania [6].

Among the members of the *An. funestus *group, resistance to insecticides has been confirmed only in *An. funestus s.s. *[7,8]. Effective control of this species requires accurate identification methods to discriminate it from the non-vectors and historically this was performed using morphological and cytogenetic methods. Morphological identification utilizes unique characteristics in life stages of the mosquitoes and requires eggs from wild caught females to be reared through the different life forms. This takes about four to six weeks and there is a chance of misidentification because the species within the group show overlapping characteristics [1]. Cytogenetic analysis of giant polytene chromosomes has also been used for species identification after studies revealed species-specific chromosomal banding patterns resulting from fixed paracentric inversions, and published chromosomal maps now exist for *An. funestus s.s., An. parensis*, *An. leesoni *and *An. rivulorum *[9,10]. However, this technique involves the extraction of ovarian nurse cells from semi gravid females, thereby limiting its application to this life stage, which means that males and immatures cannot be identified. In addition, *An. funestus s.s. *can not be discriminated from *An. vaneedeni *in situations where these species are homozygous for inversions on chromosome arm 2 [10].

Over the last decade these older identification methods have been largely superseded by more rapid DNA-based molecular approaches based on PCR. The first of these was an allele-specific polymerase chain reaction (AS-PCR) assay, which identified *An. leesoni, An. vaneedeni, An. rivulorum *and *An. funestus s.s. *by exploiting species-specific polymorphisms in the ribosomal DNA gene (rDNA) [11]. However, this method is unable to distinguish *An. parensis *from *An. vaneedeni *and, therefore, an improved AS-PCR protocol was developed to detect and discriminate all five species and this has rapidly become the 'gold standard' for identification of members of the species group [12]. Disadvantages of the current PCR approach include the requirement for post-PCR processing (gel electrophoresis of PCR products) and manual scoring of test samples which can be prone to error due to the similar amplicon sizes generated by certain species.

The aim of the present study was to develop a real-time PCR-based method for identification of members of the *An. funestus *group that overcomes the disadvantages of the previously described methods and is as sensitive as the gold standard AS-PCR approach. These aims were addressed by developing high-throughput 'closed-tube' approaches based on Melt Curve Analysis (MCA), High Resolution Melt (HRM) and TaqMan SNP genotyping.

## Methods

### Samples and DNA extraction

For the initial optimization of each assay and for the blind trial, field-caught mosquito specimens of *An. leesoni, An. vaneedeni, An. rivulorum, An. parensis *and *An. funestus s.s. *were collected from Ghana, South Africa and/or Mozambique. DNA was extracted from single mosquitoes using either the Livak or Collins methods [13,14]. Using these methods DNA yield from a single mosquito was typically 2-5 μg (as determined by absorption at 260 nm using a NanoDrop spectrophotometer, NanoDrop Technologies). These samples had been initially identified to species at the time of collection using morphology and AS-PCR [12]. The blind species identification trial was performed using 96 samples, which included a range of the above species and a number of negative controls. The quality and quantity of DNA obtained from these specimens varied considerably and many had been subject to repeated freeze-thawing so the trial represented a thorough test of the robustness of each assay. To determine the sensitivity of the three identification methods they were further tested using a dilution series of DNA from each of the five species in the *An. funestus *group. For this, DNA preparations were diluted to 20 ng/μl and then serially diluted down to a 1 in 1 × 10^6 ^dilution.

### AS-PCR

AS-PCR was performed according to the protocol described previously [12] with minor modification. PCR was performed in a final volume of 14 μl containing 6 μl of ReddyMix PCR master mix (Thermo fisher scientific, UK) and 0.24 μM of each primer. The thermal cycling conditions were unchanged.

### MCA assay

The MCA assay utilized the universal forward and species-specific reverse primers of Koekemoer *et al *[12] with the exception of the VAN and FUN primers where new species-specific primers (VAN3 and FUN1) were designed in order to generate amplicons of an optimal melting temperature (Table 1). PCR and subsequent melt curve analysis was carried out using the Rotor-Gene 6000 (Corbett Research). PCR reactions (20 μl) consisted of 1 μl genomic DNA, 10 μl of SensiMix™ kit (Quantace), 0.4 μl SYBR Green 1 (Quantace) and 250 nM of each primer (UV, FUN1, LEES, VAN3, PAR and RIV). Cycling conditions consisted of one cycle of 95°C for 10 minutes followed by 40 cycles of 95°C for 15 seconds, 55°C for 30 seconds and 72°C for 30 seconds. This was immediately followed by a melt step of 72-95°C rising by 1°C and holding for 90 seconds for pre-melt conditioning for the first step and subsequently 5 seconds for each step afterwards. The increase and decrease in fluorescence of SYBR Green during PCR and the melt phase, was acquired on the green channel (470 nm excitation and 510 emission) of the Rotor-Gene.

**Table 1:** Primers and probes used in the identification of *Anopheles funestus *species using real time assays (MCA, HRM and TaqMan)

Name	Primer /probe	Reporter Dye	Sequence (5'-3')	Quencher
FUN1	Primer	-	GGCATCGATGGGTTAATCATG	-
VAN1	Primer	-	AAACCCCAAGATGTGCTCC	-
				
VAN3	primer	-	GGTTTTCAAATGAATCTC	-
N PAR	Primer	-	ATACTTGTGTGTGTGTGTATTTG	-
RIV TM	probe	**Cy5**	CTATGGCGAGACCCCGTCTAGTG	BHQ2^a^
				
FUN TM	probe	**ROX**	CATGGGGAAATTCAATCGAAAACCTCT	BHQ2^a^
				
PAR TM	probe	**VIC**	CGG AAC CTA GCT TGG	MGBNFQ^b^
VAN TM	probe	**Quasar 705**	CGT TGT GAA AAA TGG AGA TTC ATT TGA AAA CC	BHQ2^a^
				
LEES TM	probe	**6-FAM**	CCG ACC GAT GTA CA	MGBNFQ^b^

^a^BHQ2 (black hole quencher 2)

^b^MGBNFQ (minor groove binder non-fluorescent quencher)

### HRM

The HRM assay also utilized the universal forward and species-specific reverse primers of Koekemoer *et al *[12]. PCR reactions (25 μl) contained 12.5 μl SensiMix HRM kit (Quantace), 200 nM of each primer; 1 μl Eva Green dye, 1 μl genomic DNA. Samples were run on the Rotor-Gene 6000 using the temperature cycling conditions of: 1 cycle of 95°C for 10 minutes; 40 cycles of 95°C for 15 seconds, 56°C for 30 seconds and 72°C for 30 second. This was immediately followed by a melt step of 77-94°C rising by 0.1°C and holding for 90 seconds for pre-melt conditioning for the first step and subsequently 2 seconds for each step afterwards. Changes in fluorescence of Eva Green during PCR and the melt phase were monitored on the green channel (470 nm excitation and 510 emission) of the Rotor-Gene.

### TaqMan

Nucleotide alignments of ribosomal DNA (rDNA) gene sequences of the different species of *An. funestus *available in the National Center for Biotechnology Information (NCBI) database revealed species-specific sequences allowing the design of discriminating probes. Unfortunately there was no region of conserved sequence to design a common reverse primer for the TaqMan assay. Therefore the universal forward and species-specific reverse primers of Koekemoer *et al *[12] were utilized with the exception of the original primers specific for *An. parensis *and *An. vaneedeni *where two new species specific reverse primers were designed (new PAR and VAN1, Table 1). Each species-specific probe was designed to anneal to sequence between the corresponding species-specific reverse primer and the universal forward primer. Probes designed to anneal over sequence containing two or less species-specific SNPs were designed as minor groove binding (MGB) probes (Applied Biosystems) using the Primer Express™ Software Version 2.0 (Applied Biosystems). The minor groove binder provides more accurate allelic discrimination by increasing the T_M _between matched and mis-matched probes [15]. Probes designed to anneal over sequence containing more than two species-specific SNPs were designed manually and synthesized as dual-labeled probes by Thermo Fisher Scientific or Biosearch Technologies. The sequence and quencher modifications of each probe are shown in Table 1. The five probes were labeled at the 5' end with VIC (probe PAR TM) for *An. parensis *detection, 6FAM (probe LEE TM) for *An. leesoni *detection, Cy5 (probe RIV TM) for *An. rivulorum *detection, ROX (probe FUN TM) for *An. funestus s.s. *detection and Quasar 705 (probe VAN TM) for *An. vaneedeni *detection. These five flurophores have distinct emission and excitation spectra allowing their independent detection in a single reaction.

PCR reactions (20 μl) contained 10 μl of SensiMix DNA kit (Quantace), 200 nM of each probe, 1 μM each of UV, FUN and VAN 1 primers, 0.5 μM of RIV, LEES, New PAR primers and 1 μl of DNA template. Reactions were run on the Rotor-Gene 6000 using the temperature cycling conditions of: 10 minutes at 95°C followed by 35 cycles of 95°C for 15 seconds and 60°C for 60 seconds. The increase in VIC, FAM, ROX, CY5 and Quasar 705 fluorescence was monitored in real time by acquiring each cycle on the yellow (530 nm excitation and 555 nm emission), green (470 nm excitation and 510 emission), orange (585 nm excitation and 610 nm emission), red (625 nm excitation and 660 nm emission) and crimson channels (680 nm excitation and 710 nm emission) of the Rotor-Gene respectively.

## Results

### AS-PCR

The sensitivity of the AS-PCR assay was evaluated using serially diluted DNAs from each species of mosquito. The detection limit of each of the five species was a 1 in 500 dilution, which represents 0.04 ng of DNA. The results from the blind species identification trial using the PCR method are shown in Table 2. The AS-PCR method showed a low failure rate (no amplification in PCR) and did not incorrectly identify any samples.

**Table 2:** Performance of four assays in the *Anopheles funestus *group species identification blind trial

	AS-PCR	MCA	HRM	TaqMan
Correct scores	90	84	88	89
Failed reactions	6	4	2	7
Miscored	0	8	6	0

### MCA assay

The melt-curve real-time PCR assay uses allele-specific primers to generate products of different length and/or GC content that are discriminated by the different melt temperature of the PCR amplicons. PCR in the presence of the intercalating dye SYBR green is followed by a melt step which results in denaturisation of the PCR products and a resulting decrease in SYBR green fluorescence as the dye is released. A plot of the negative first derivative of the collected fluorescence against temperature results in melt peaks with characteristic melting temperatures (T_M_).

**Figure 1:**
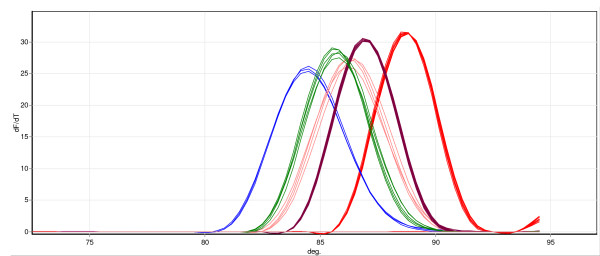
**Identification of members of the *An. funestus *group by melt-curve real-time PCR analysis**. In this example five to seven specimens of *An. rivulorum *(red trace), *An. leesoni *(blue trace), *An. funestus *(brown trace), *An. parensis *(green trace) and *An. vaneedeni *(pink trace) were tested. A plot of negative first derivative of the collected fluorescence against temperature results in melt peaks with characteristic melting temperatures (T_M_).

After optimization using DNA templates of known species the MCA was able to detect and discriminate *An. leesoni, An. vaneedeni, An. rivulorum, An. parensis *and *An. funestus s.s. *Figure 1 depicts the characteristic melt curves produced by the five different mosquito species after plotting a negative first derivative of fluorescence against temperature. The average T_M _for 10 samples per species was determined and the results are presented in Table 3. During optimization it became clear that it was sometimes difficult to differentiate *An. vaneedeni *from *An. parensis *and *An. funestus s.s. *specimens as the melt-temperatures of the amplicons produced from each species was similar. Attempts were made to improve the discrimination of these samples by designing alternative primers (to generate smaller or larger amplicons with different T_M_) for the detection of *An. vaneedeni *and *An. funestus s.s. *but the primers described in the methods ultimately gave the clearest discrimination of these species. This issue affected the performance of this assay in the species identification trial (Table 2) where the MCA exhibited a low failure rate but incorrectly identified a number of samples. In most instances this was due to misidentification of *An. vaneedeni*, *An. funestus s.s. *and *An. parensis *specimens. Analytical sensitivity of the MCA assay was investigated using a dilution series of DNAs for each of the five species and the detection limit was found to be a 1 in a 1,000 dilution, which represents 0.02 ng of DNA.

**Table 3:** Average melt curve T_M _and standard deviation values for different members of the *An. funestus *group.

Species	Average T_M_	Standard deviation (±)
*An. rivulorum*	88.71	0.087
*An. Leesoni*	84.69	0.34
*An. vaneedeni*	86.17	0.6
*An. Funestus*	87	0
*An. Parensis*	85.82	0.175

### HRM

High Resolution Melt (HRM) is an extension of the MCA assay but employs next generation real-time PCR thermocyclers with high thermal and optical precision. HRM analyses involve the PCR amplification of DNA containing the SNP(s) of interest in the presence of a third generation fluorescent dsDNA dye. The new generation of dyes for this purpose such as SYTO 9 (Invitrogen), LC Green (Idaho Technologies) and Eva Green (Biotium Inc) are less inhibitory to PCR than traditional dyes, which allow them to be used at higher concentration to achieve maximum saturation of the resulting dsDNA amplicon. A high-resolution melt step is then performed, centered around the T_M _of the amplicon. As the dsDNA dissociates into single strands the dye is released and the fluorescence diminishes giving a melt curve profile characteristic of the sequence of the amplicon [16].

**Figure 2:**
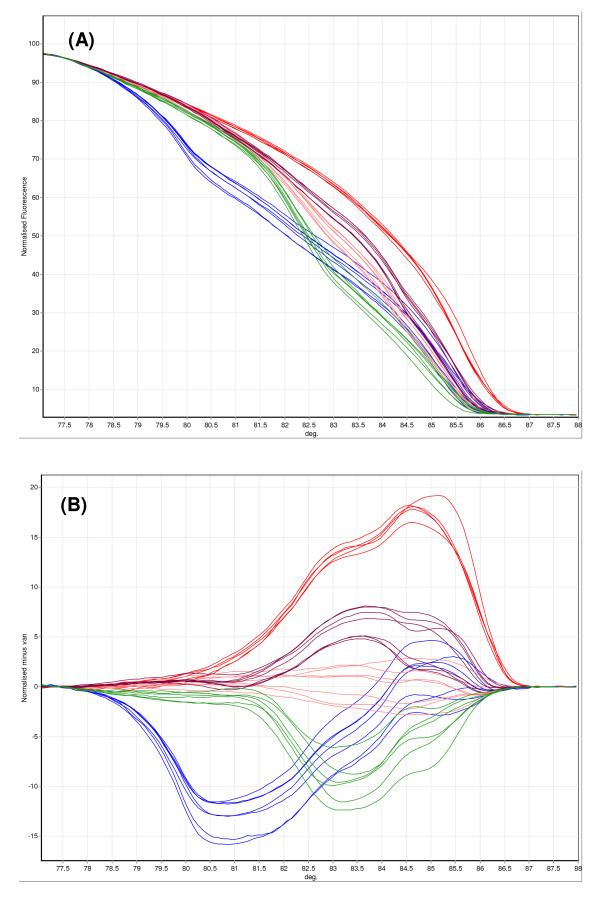
**Identification of members of the *An. funestus *group by High Resolution Melt analysis**. In this example, five to seven specimens of *An. rivulorum *(red trace), *An. leesoni *(blue trace), *An. funestus *(brown trace), *An. parensis *(green trace) and *An. vaneedeni *(pink trace) were tested. A) Normalized melt curve for different *An. funestus *species. B) Difference plot for samples as in (A).

After optimization the HRM assay was able to discriminate all five target species of the *An. funestus *group from the melting temperature of the PCR amplicons which ranged in size from 146 to 587 bp. Samples were scored by examining normalized and difference melt plots using the associated Rotor-Gene Software (version 1.7). Figure 2, shows a normalized plot as well as a difference plot for several specimens of each species of mosquito. The difference plot generated from the normalized data highlights differences between a selected genotype and the other samples. The sensitivity of the HRM assay was evaluated using a dilution series of DNA for each species. The detection limit of each of the five species was a 1 in a 1,000 dilution, which represents 0.02 ng of DNA. When the performance of the HRM assay was assessed in a blind trial using DNA extracted from wild-caught mosquitoes (Table 2) it displayed a low failure rate but like the MCA assay incorrectly identified a number of samples. This included the misidentification of two water negative controls (as *An. funestus s.s. *and *An. rivulorum*), which may indicate a potential problem with background/non-specific fluorescence in this assay.

### TaqMan

The TaqMan assay is a PCR method which uses oligonucleotide probes that are dual-labeled with a fluorescent reporter dye and a quencher molecule. Amplification of the probe-specific product causes cleavage of the probe, generating an increase in reporter fluorescence as the reporter dye is released away from the quencher. By using different reporter dyes, cleavage of allele-specific probes can be detected in a single PCR.

**Figure 3:**
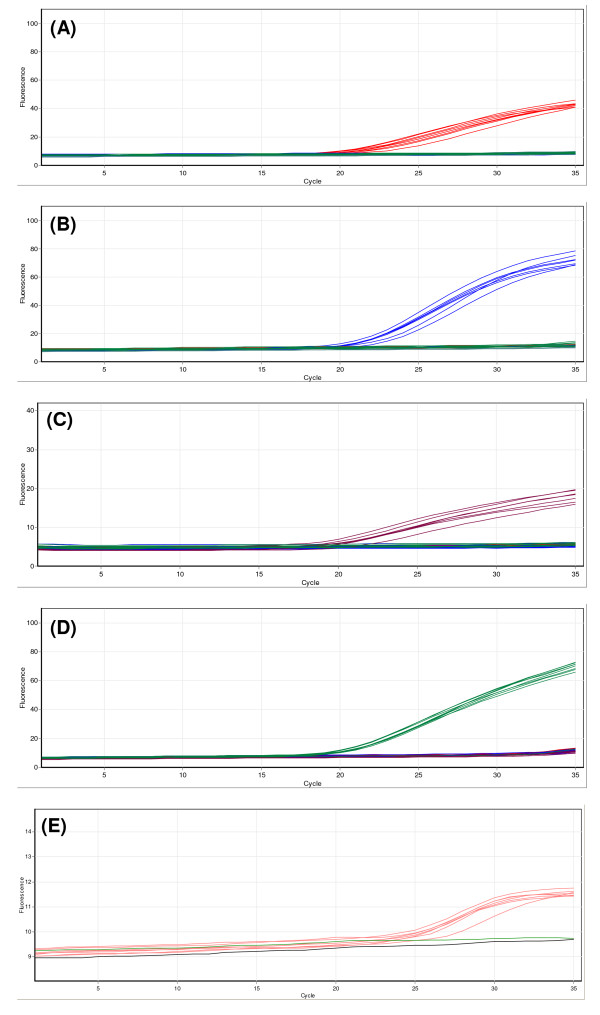
**Identification of members of the *An. funestus *group using the multiplex TaqMan assay**. Seven to ten specimens of *An. rivulorum *(red trace), *An. leesoni *(blue trace), *An. funestus *(brown trace), *An. parensis *(green trace) and *An. vaneedeni *(pink trace) were tested. (A) Cycling of the RIV probe (Cy5 labelled), (B) cycling of the LEES probe (6FAM labelled), (C) cycling of the FUN probe (ROX labelled), (D) cycling of the PAR probe (VIC labelled) and (E) cycling of the van probe (Quasar 705 labelled).

After optimization the multiplex TaqMan assay effectively identified control templates of the five members of the *An. funestus *group. Figure 3 shows an example of using this assay for the identification of seven to ten samples of each species. An increase in the fluorescence of Cy5 (probe RIV), 6FAM (LEES probe), ROX (FUN probe), VIC (PAR probe) and Quasar 705 (VAN probe) identifies *An. rivulorum, An. leesoni*, *An. funestus s.s.*, *An. parensis *and *An. vaneedeni *specimens respectively. An increase in two or more of the dyes would indicate a hybrid or a contaminated sample. During optimization it was noticed that the fluorescent signal generated by specific binding of the VAN probe was significantly lower than the other probes as quantified in relative fluorescent units. The exact reason(s) for this is unknown, however, amplification of the specific *An. vaneedeni *amplicon was acceptable (when checked by running products on agarose gel electrophoresis) and replacement of the fluorescent dye label and/or redesign of the probe did not improve the fluorescent signal generated. Despite the lower fluorescent signal it was still possible to effectively identify *An. vaneedeni *specimens by using the auto-scale function of the associated Rotor-Gene software. The TaqMan PCR was originally run for 40 temperature cycles, however, low level non-specific amplification was occasionally observed from cycling of the LEES (6FAM-labelled) and PAR (VIC-labelled) probes specific for *An. leesoni *and *An. parensis *respectively after 35 cycles. This was successfully eliminated by restricting the number of temperature cycles to 35.

The analytical sensitivity of the TaqMan assay was assessed using serially diluted DNAs from the five representative species. The detection limit of each species was a 1 in a 1,000 dilution, which represents 0.02 ng of DNA. The performance of the TaqMan assay in the blind trial using DNA extracted from wild-caught mosquitoes (Table 2) was found to be comparable to AS-PCR with a low failure rate and no incorrectly identified samples.

## Discussion

In this study, protocols were developed for three high-throughput multiplex real-time PCR assays (TaqMan, HRM and MCA) for the identification of members of the *An. funestus *group that occur commonly throughout much of Africa. Unlike conventional AS-PCR, these assays do not require processing of samples by agarose gel electrophoresis, which is time consuming, restricts throughput and requires the use of the safety hazard ethidium bromide.

Of the three fluorescent assays, the MCA assay held promise for being the most cost-effective as it employs standard oligonucleotide primers, has no requirement for fluorescently labelled probes and can be run on single channel real-time PCR machines. Analysis of one sample costs approximately US $0.65 using this method. The assay developed in this study was able to detect and discriminate all five target species using DNA templates of known concentration and was more sensitive than the standard AS-PCR. However, when the assay was tested in a blind trial using DNA extracted from wild-caught mosquitoes of variable concentration and quality it became evident that this assay occasionally misidentifies *An. vaneedeni*, *An. funestus s.s. *and *An. parensis *specimens. This is likely due to the relatively close T_M _of the specific amplicons produced during amplification of each species (Table 3). For MCA it is recommended that the amount of DNA template used in PCR is consistent between samples as large differences in starting template will affect the observed T_M_. It is, therefore, possible that this assay could be improved if DNA concentration was adjusted. However this constitutes an additional step in the protocol and would require DNA quantification using a spectrophotometer or gel electrophoresis. It is likely that the chances of misidentification will be reduced when the assay is used to test mosquitoes collected from much of Sub-Saharan Africa where *An. vaneedeni *does not occur due to the limited distribution of this species. This should make scoring the other species using MCA easier as the gap in T_M _between amplicons generated from *An. funestus s.s. *and *An. parensis *specimens will be clearer.

To see if a more recent melt-curve approach could more accurately identify the small difference in T_M _between these three species an assay based on HRM was developed. Like MCA the running cost of HRM is low (US $0.65 per sample) as it uses standard oligonucleotide primers and a cheap intercalating dye; however the disadvantage of this platform is the capital cost required for the more expensive real-time PCR machine required. The HRM method showed promise during optimization with templates of known genotype (where DNA concentration was adjusted to be consistent for all samples) with a sensitivity greater than AS-PCR, but, like the MCA assay, subsequently performed less well in the blind genotyping trial. This is likely explained by variable DNA quality and quantity among the samples tested, leading to some samples amplifying after ~30 cycles or failing to reach full plateau phase. In addition the HRM method incorrectly identified two negative control samples as *An. leesoni *and *An. rivulorum *indicating a potential problem with background/non-specific fluorescence.

In order to overcome the problems with the two melt-curve based approaches, an assay based on TaqMan SNP genotyping was developed. This approach has proven to be very sensitive and robust in the detection and discrimination of the *Plasmodium *species responsible for human malaria, members of the *An. gambiae *complex that vector the disease and for detection of mutations in the mosquito genome that confer insecticide resistance [17-20]. In tests of analytical sensitivity and in the blind trial the TaqMan assay was shown to have a specificity and sensitivity at least as good as the standard AS-PCR. Of the three fluorescence assays tested in this study, this assay was the only method to record no misidentification of samples indicating the robustness of this platform. The unambiguous identification of samples by the TaqMan assay may in part be due to the dual layer of specificity provided by the combination of both allele-specific primers and allele-specific probes. The running cost of the TaqMan assay, as performed in this study, is slightly higher than MCA and HRM as it uses fluorescently-labelled probes (US $0.95 per sample). Additional experiments were carried out to see if reagent volumes could be reduced without affecting the sensitivity of the assay. With the real-time PCR machine used in this study (Rotor-Gene 6000™, Corbett Research) no loss of sensitivity was observed for half volumes of reagents which reduces the running cost to US $0.55 per sample. In addition, to reduce the costs further the end-user may wish to consider selecting which probes to use in PCR based on the area where the mosquito specimens to be tested are collected, for example, it may not be necessary to test for *An. vaneedeni *in many Sub-Saharan countries as its distribution is limited to a localized region in northern South Africa. The one disadvantage of the TaqMan assay is the initial cost of the real-time PCR machine required [18]. However, the price of real-time PCR machines are falling and in the future is likely to become closer to standard thermocyclers.

## Conclusion

Of the new assays tested in this study, the TaqMan assay proved to be the most robust. The assay uses fluorescently-labeled probes with distinct emission and excitation spectra allowing the independent detection of five members of the *An. funestus *group in a single reaction. This method is at least as sensitive and specific as the gold standard AS-PCR approach and because it has no requirement for post-PCR processing is simpler to run and capable of higher throughput.

## Competing interests

The authors declare that they have no competing interests.

## Authors' contributions

SBV helped to design the assays, optimized and tested all the assays and helped draft the manuscript. CB designed the assays and drafted the manuscript. MP helped to design, optimize and test the TaqMan assay and helped review the manuscript. MSW, LMF, MC, helped draft and critically review the manuscript. LLK conceived the project and helped draft and critically review the manuscript. All authors read and approved the final manuscript.
